# A Case of Compound Fracture

**Published:** 1803-10-01

**Authors:** C. Simpson

**Affiliations:** Skipton


### *A* Case *of* Compound Fracture; *communicated by Mr.* C. Simpson, *of Skipton.*

MR. EVANS, æt. 46, of a very irritable habit, had the misfortune to fracture his leg, by a fall from a causeway, on the 8th of April last. The family surgeon was immediately sent for, who laid the leg extended upon a pillow and applied a solution of the aq. acet. lyth. by means of linen cloths. Considerable swelling of the whole limb, with violent pain and starting, came on the following day, which continued to increase until the 13th, when I first saw him. I found him in the following condition: Pulse 130, very weak and fluttering, tongue covered with a dry brown fur, constant low delirium, skin hot and parched, bowels regular, urine of high colour without sediment, and small in quantity; particular wild look, picking the bed-clothes; the leg and thigh greatly swelled, of a livid colour, soft and flaccid, so as to retain the impression made by the fingers; a great number of vesications, full of yellowish ichor, surrounding the fractured part; the broken end of the tibia protruding without the skin two inches; very restless, and with great difficulty kept in bed. I immediately laid the patient upon his side, with the knee bent as much as possible; after moderately extending the limb, the protruding end of the bone disappeared; it was then laid upon an even pillow with a splint under it, and sprinkled over with crude sal. ammon. in fine powder; afterwards covered with a large stale beer poultice, in which was mixed a portion of the same powder; the bed, &c. being adjusted, a person was directed to sit by the bed side to keep the knee steady, and the following powder to be given every two hours, well mixed in a little gruel:

℞. Mosch. chin. camph. aa. gt. xv. ammon. pp. gt. x. m. ft. pulv. and washed down with three table spoonsful of the following mixture:

℞. Confect. arom. ʒiiß. sp. cinn. sy. com. aa. ℥j. tinct. opii gt. xc. aq. puræ ℥vj. m. ft. mist. Gruel, with a good portion of red wine to be given as diet, and when thirsty to have a little wine and water.

14th, mane. A few hours after I left him last night he became quite furious, rose up in bed, tore the poultice, &c. from his leg, and on my arrival this morning, one o'clock, found him sitting upon the edge of the bed with his leg hanging down, and the end of the bone pushed out as before; the colour changed to a much darker hue, with every every other symptom of the rapid approach of mortification. I again replaced him in bed, bent the knee, and reduced the protruding bone as before; then applied the powdered sal. amm. and poultice, as usual; also, a broad belt over his breast, and firmly fixed down to each side of the bed, and gave him gt. lx. more of the tinct. opii in a dose of the cardiac mixture, and directed the powders, &c. to be regularly continued.——Vespere. Had a few hours sleep after I left him this morning, during which a profuse perspiration broke out, which continues; the delirium yet present; pulse 120; of better strength, tongue a little moist upon the edges, urine in much larger quantity; starting of the limb not so frequent; wound made by the bone filled with a black slough; no more vesications; swelling much the same; has taken his medicines well; much better to manage since the application of the belt. The wound to be dressed with the ung. res. flav. and a little of the bals. canad. mixed with it, and applied warm: two ounces of strong camphorated spirit to be applied over the whole of the limb, afterwards the powdered sal. ammon. and poultice as before; the dose of the musk and camp. to be increased to ℈j. each, with the amm. ppt. and cardiac mixture as usual; likewise to have a draught with 50 drops more tinct. opii, h. s. and a large blister to be applied between the shoulders.

15th, mane. After a sound sleep of six hours he awaked perfectly sensible; pulse 86, of good strength, perspiration copious; considerable quantity of loaded urine made since last visit; tongue moist, little or no thirst; the bulk of the leg greatly diminished and of a much brighter colour, a slight redness appearing round the edges of the wound; complains of smarting sensation when the camphor is applied; has taken medicines regularly; blister rises well; no stool since the 13th. The powders and mixture to be discontinued, to have a solution of the neutral salts, with an infusion of senna; the wound to be dressed as before, and poultice changed for white bread and milk.—Vespere. Has had two stools from the solution, leg perfectly easy, and remains in a good position; swelling continues on the decrease; in good spirits, pulse 80, perspiration more moderate; a little matter discharges from the wound; to be dressed with balsam and poultice as before; to have a solution of cream tartar. for common drink, and a draught at bed time with 40 drops tinct. opii.

17th. Continues convalescent; wound discharges freely; an eighteen tail'd bandage applied, with another long splint splint upon the inside of the leg; to have a nourishing diet with a pint of red-port daily, the leg to remain in a relaxed position.

June 14. Until this time has continued to recover daily; the wound discharged freely until the slough digested out, afterwards granulated and healed in the most favourable manner; is now able to walk a little upon crutches.

July 22. Is now able to walk with the assistance of a stick; the leg equally as straight and of the same length as the other.

If the favourable termination of this case lead to a further trial of the conjoined use of *large doses* of musk, camphor, opium, and ammonia, together with the external use of crude sal. ammoniac, and with similar effects, I shall consider myself highly gratified: there cannot be a doubt of the mortification here being arrested by steady perseverance in the above plan, assisted by strict attention to the relaxed position.

The whole limb exhibited every appearance of gangrene, having actually taken place when I first saw him, which demanded the most active and speedy treatment, and which hurried rapidly forward before the medicines could arrive, being seven miles distant from my place of abode. I consider *musk, opium,* and *ammonia,* the sovereign remedy in those cases, provided they be given *in full doses,* and at *short intervals.* Nothing can more completely contradict the impropriety of tight bandages in all cases of fracture than this. Mr. Corner, the gentleman first called in (since deceased) applied a circular linen roller firmly round the fractured part, afterwards the solution mentioned. To the tight application of the roller, the extended position the limb was kept in, and the patient's habit of body at the time, I attribute the attack of mortification.

*August* 4, 1803.

